# Comparing contamination rates in paired blood culture sets with a single-time stamp vs multiple-time stamps

**DOI:** 10.1017/ash.2025.10103

**Published:** 2025-09-16

**Authors:** Abhilasha Borad, Ali Ibrahim, Susan E. Boruchoff, Keith S. Kaye, Ahmed Abdul Azim

**Affiliations:** 1 Beth Israel Deaconess Medical Center, Boston, MA, USA; 2 Rutgers, The State University of New Jersey, New Brunswick, NJ, USA; 3 Division of Allergy, Immunology and Infectious Diseases, Rutgers Robert Wood Johnson Medical School, New Brunswick, NJ, USA

## Abstract

This retrospective analysis compares the recovery rate of commensal organisms in two sets of blood cultures with a single-time stamp (STS) versus ones with multiple-time stamps (MTS) in an academic tertiary center. Rates in which both sets were positive for commensals were numerically higher in STS versus MTS.

## Introduction

The current recommendations regarding blood culture (BC) methods specify that optimal BC sampling in adults consists of collecting 20–30 mL of blood per culture set, evenly distributed between aerobic and anaerobic bottles, with two sets (totaling 40–60 mL) obtained from separate venipuncture sites, collected sequentially.^1,2^ Improper sampling methods increase the risk of BC contamination, leading to unnecessary hospital admissions, inappropriate antibiotic therapy, prolonged lengths of stay, and increased healthcare costs.^3,4^

Clinicians at our academic tertiary center, Robert Wood Johnson University Hospital, noticed repeated instances where two sets of BCs obtained in the Emergency Department (ED) with identical collection time stamps in the Electronic Medical Record (EMR) grew commensal organisms in both sets in patients with low pretest probability for true bacteremia. The policy at our hospital is to obtain BC paired sets from different peripheral sites when possible. We hypothesized that these instances where two BC sets had the same time stamp indicated that blood was obtained from a single stick at one anatomic site and inoculated into both sets of BC bottles, rather than from two separate sticks and anatomic sites. This assumption was supported in the ED setting based on conversations with ED staff and attendings, as well as various patients over the years who reported that four BC bottles were all drawn from a newly placed peripheral intravenous (IV) catheter.

We evaluated the recovery rate of commensal organisms in both sets of BCs, predicting that rates would be higher in the single-time stamp (STS) BC group compared to the multiple-time stamps (MTS) group.

## Methods

This is a single-center retrospective analysis using data from clinical microbiology and hospital databases. The primary study outcome was the rate of recovery of predefined commensals^5^ (see Table 1), which were compared for patients with two sets of BCs drawn with identical time stamps (categorized as STS) and for patients with two BC sets drawn with different time stamps within 1 hour of one another (categorized as MTS). All paired BC sets, regardless of whether cultures were obtained peripherally or from a central line, from adult patients who presented to our 650-bed tertiary care center between June 1, 2022, and November 15, 2023, were included. Pairs of BC sets with non-commensal organisms isolated in any set were excluded. Collected data includes BC timing, BC results, and admission unit. Rates from the ED and non-ED inpatient locations (which included both intensive care unit and medical-surgical wards) were also compared. We excluded patients under 18 years of age.

**Table 1.** Predefined commensal organisms

*Actinomyces* spp.	*Staphylococcus auricularis*
*Aerococcus sanguinicola*	*Staphylococcus capitis*
*Aerococcus urinae* spp.	Coagulase-negative *Staphylococcus*
*Arthrobacter* spp.	*Staphylococcus epidermidis*
*Bacillus* spp.	*Staphylococcus haemolyticus*
*Brevibacterium* spp.	*Staphylococcus hominis*
*Corynebacterium* spp.	*Staphylococcus pettenkoferi*
*Cutibacterium acnes*	*Staphylococcus saprophyticus*
*Dermabacter* spp.	*Staphylococcus schleiferi*
*Janibacter* spp.	*Staphylococcus simulans*
*Kocuria* spp.	*Streptococcus anginosus*
*Lactobacillus* spp.	*Streptococcus lutetiensis*
*Lactococcus garvieae*	*Streptococcus mitis*
*Leptotrichia* spp.	*Streptococcus mutans*
*Micrococcus* spp.	*Streptococcus parasanguinis*
*Moraxella catarrhalis*	*Streptococcus salivarius*
*Moraxella osloensis*	Viridians Group *Streptococcus*
*Neisseria mucosa*	
*Neisseria sicca*	
*Paenibacillus* spp.	
*Propionibacterium* spp.	
*Rothia* spp.	

spp = species.

## Results

A total of 5,296 paired BC sets were evaluated, with 4,411 classified as STS and 885 as MTS. A total of 343 pairs, including 6.3% (278/4,411) of STS and 7.3% (65/885) of MTS, grew only commensal organisms in at least one of the two BC sets (Table 2). Furthermore, 28.1% (78/278) of those STS pairs and 18.5% (12/65) of those MTS pairs grew commensal organisms in both BC sets (*P* = .11) (Table 2). These findings were consistent when analyzed separately for ED and non-ED settings (Table 2).

**Table 2.** BC positivity for commensal organisms only, as a function of positive BC with single-time stamp (STS) vs multiple-time stamps (MTS) and patient location

	Single-time stamps (STS); n (% of total)	Time stamps Within 1 Hour (MTS); n (% of total)	Total; n (% of total)
**All units**			
1 of 2 BC Positive	200 (71.9)	53 (81.5)	253 (73.8)
2 of 2 BC Positive	78 (28.1)^	12 (18.5)^	90 (26.2)
Total	278	65	343
**Emergency department**			
1 of 2 BC Positive	158 (71.5)	39 (81.3)	197 (73.2)
2 of 2 BC Positive	63 (28.5)^^	9 (18.8)^^	72 (26.8)
Total	221	48	269
**Non-ED inpatient units**			
1 of 2 BC Positive	42 (73.7)	14 (82.4)	56 (75.7)
2 of 2 BC Positive	15 (26.3)^#^	3 (17.6)^#^	18 (24.3)
Total	57	17	74

Commensal definition based on microbiology laboratory data; ^ *P* = .11; ^^ *P* = .17; # *P* = .46; BC positive = blood culture positive for a commensal organism.

## Discussion

When two sets of BCs were drawn within one hour of each other, the rate at which both sets were positive for commensal organisms was higher in BCs with STS compared to BCs with MTS, although this did not reach statistical significance.

This study expands upon the literature regarding single-site versus multi-site sampling blood culture techniques. Mcleod *et al.* identified the collection of at least two BCe sets from the same site as a frequent problem in the ED, with a reported 15.13% of samples being drawn in this fashion.^6^ Yu *et al.* and Ekwall-Larson *et al.* reported no statistically significant difference in contamination between multi-site sampling and single-site sampling in Swedish EDs.^7,8^ Our study demonstrates that multi-site sampling (or BC with MTS) may yield higher rates of commensal organism culture positivity in two of two BC sets.

This analysis had limitations. Most notably, we assumed that paired BC sets with identical time stamps represent single-site sampling, which might not have been the case in all instances and would have resulted in potential misclassification bias. We also did not adjudicate whether patients had true bacteremia with commensal organisms. Patients with more comorbidities are likely to have difficult IV access, making single-site sampling BC more likely in this sicker group. Other limitations include a small sample size in a single-center, and the fact that we did not control for which person was drawing the cultures.

This study highlights the importance of utilizing optimal blood culture sampling techniques to best inform clinical decision-making and improve patient care. Although underpowered to demonstrate a statistically significant association, the clinical implication of this study is that by increasing the likelihood of recovering commensal organisms in two sets of BCs, single-site sampling practices may increase the risk of misclassifying patients as having true bacteremia, potentially leading to unnecessary antibiotic use and increased length of stay.^4^ Importantly, this study highlights the need for efficient and accurate documentation methods embedded within the EMR regarding the anatomic sites and timing of BC sampling. One way to do this is to raise awareness amongst nursing staff and phlebotomists regarding the potential adverse impact of inaccurate documentation on patient care. Moreover, collaborating with information technology staff on EMR “nudges” and streamlining documentation can be utilized to improve blood culturing processes.

## References

[1] Miller JM , Binnicker MJ , Campbell S , et al. Guide to utilization of the microbiology laboratory for diagnosis of infectious diseases: 2024 update by the Infectious Diseases Society of America (IDSA) and the American Society for Microbiology (ASM). Clin Infect Dis 2024,ciae104. 38442248 10.1093/cid/ciae104

[2] Sautter RL , Parrott JS , Nachamkin I , et al. American Society for Microbiology evidence-based laboratory medicine practice guidelines to reduce blood culture contamination rates: a systematic review and meta-analysis. Clin Microbiol Rev 2024;37:e00087-24. 10.1128/cmr.00087-24 PMC11629619 39495314

[3] Halverson S , Malani PN , Newton DW , *et al.* Impact of hourly emergency department patient volume on blood culture contamination and diagnostic yield. J Clin Microbiol 2013;51:1721–1726. 23515549 10.1128/JCM.03422-12 PMC3716046

[4] Gander RM , Byrd L , DeCrescenzo M , *et al*. Impact of blood cultures drawn by phlebotomy on contamination rates and health care costs in a hospital emergency department. J Clin Microbiol 2009;47:1021–1024. 19171686 10.1128/JCM.02162-08 PMC2668314

[5] Buetti N , Marschall J , Drees M , et al. Strategies to prevent central line-associated bloodstream infections in acute-care hospitals: 2022 update. Infect Control Hosp Epidemiol 2022;43:553–569. 35437133 10.1017/ice.2022.87 PMC9096710

[6] McLeod CG. Reducing blood culture contamination in the emergency department. J Nurs Care Qual 2020;35:245–251. 32433148 10.1097/NCQ.0000000000000441

[7] Yu D , Larsson A , Parke Å , et al. Single-sampling strategy vs. Multi-sampling strategy for blood cultures in sepsis: a prospective non-inferiority study. Front Microbiol 2020;11:1639. 32793149 10.3389/fmicb.2020.01639 PMC7390949

[8] Ekwall-Larson A , Yu D , Dinnétz P , et al. Single-site sampling versus multisite sampling for blood cultures: a retrospective clinical study. J Clin Microbiol 2022;60:e01935-21. 34851687 10.1128/JCM.01935-21 PMC8849186

